# The cognitive and psychosocial effects of online learning in medical students during and after the COVID-19 pandemic: a mixed-methods study from Karachi, Pakistan

**DOI:** 10.1186/s12909-025-07334-0

**Published:** 2025-07-01

**Authors:** Asad Saulat Fatimi, Syeda Sadia Fatima, Russell Seth Martins, Rafay Iqbal, Saniya Sabzwari

**Affiliations:** 1 Medical College, Aga Khan University, Karachi, Sindh Pakistan; 2 Department of Biological and Biomedical Sciences, Aga Khan University, Karachi, Sindh Pakistan; 3 Clinical and Translational Research Incubator (CITRIC), Aga Khan University, Karachi, 74800 Pakistan; 4 Department of Family Medicine, Aga Khan University, Karachi, Sindh Pakistan

**Keywords:** Cognitive, Medical education, Online learning, Pedagogy, Psychological, Social

## Abstract

**Background:**

The COVID-19 pandemic precipitated a swift shift towards e-learning platforms in medical education. While research has predominantly concentrated on evaluating the efficacy and obstacles associated with online learning, the cognitive and psychosocial dimensions within medical education remain largely underexplored.

**Methods:**

A mixed-methods explanatory sequential study was conducted from July 2021 to February 2022 amongst medical students engaged in online learning for at least six months in Karachi, Pakistan. In this study, participating medical students were requested to reflect on their experiences with and perceptions of online learning during and after the pandemic. The quantitative phase of the study involved a digital questionnaire, while the qualitative phase of the study employed semi-structured focus group discussions (FGDs). Quantitative data were summarized using descriptive statistics and categorical associations were statistically tested using Pearson’s chi-squared (𝜒^2^) test. The transcribed FGDs were coded using a scissors-and-sort technique and qualitatively summarized in a narrative fashion and in descriptive memoranda.

**Results:**

A total of 262 students were included in the quantitative arm of the study (of whom 18 were included in the qualitative arm). 46.9% of the students were males, 51.1% were pre-clinical students, and 13.4% belonged to universities aside from Aga Khan University. Participating students reported that their academic screen time rose dramatically during the COVID-19 pandemic, with 80.5% spending over 3 h on screens, compared to 52.3% engaging for less than 1 h pre-pandemic. Online learning showed significant gender-based impacts: male students felt less engaged (*p* = 0.007), while female students reported less motivation (*p* = 0.007) and anxiety reduction (*p* = 0.042). Duration of online exposure influenced outcomes; students with less screen time for learning reported better self-regulation (*p* = 0.019), whereas higher entertainment screen time correlated with poorer achievement of learning objectives (*p* = 0.019). Pre-clinical students reported that they faced more challenges with engagement (*p* = 0.038), but were less likely to feel exhausted (*p* = 0.001) or have trouble paying attention during online studies than their clinical counterparts (*p* = 0.007). Overall, while some students highlighted the efficiency and convenience of online learning, many observed that they experienced increased distraction and isolation, suggesting a mixed impact on educational outcomes.

**Conclusion:**

Online medical education offers opportunities and challenges, with our results suggesting clear cognitive and psychosocial effects. Balancing student needs with hybrid learning methods is necessary to implement online learning pedagogies effectively.

## Introduction

In today’s era of rapid technological advancement, technology profoundly influences medicine and medical education. While medical education has many long-established pedagogical approaches to learning, which include the conventional face-to-face (FTF) approach, constantly improving technologies and an unprecedented degree of digital connectivity have allowed for a shift from traditional forms of teaching to other media which employ online, distance, or electronic learning [1].

Although the ubiquity of more traditional teaching methods in medical education has led to a reluctance in adopting these newer practices, the COVID-19 pandemic created a paradigm shift towards online modalities to minimize disruptions in learning [2]. While the pandemic is officially no longer a public health emergency as per the World Health Organization (WHO), it has prompted educators to explore and appreciate the potential of online learning, leading to its continued integration into medical curricula.

The benefits of online learning are numerous, and include flexibility, the lack of a need to commute, and being able to learn at one’s own pace [3–5]. In contrast, generalizable barriers to implementing online learning include factors such as family distractions, difficulties in concentrating and internet connectivity issues, while more medicine-specific barriers include the irreplaceability of in-person clerkships for clinical skill acquisition [2, 3, 6].

Exploring the efficacy, detriments, and general perceptions associated with online learning is crucial in determining its feasibility as instructional models in medical education rely heavily on social constructivism e.g. problem-based learning (PBL), team-based learning (TBL), and competency-based education [7, 8]. As the phenomenon of mainstreaming online learning in medical education continues, its impact on the tenets of social constructivism needs exploration, including psychosocial and cognitive domains [6].

While some studies exist on the psychological impact of COVID-19 on medical students, there is little literature that focuses on this aspect of online learning outside of the pandemic era [9, 10]. This paucity of literature is especially pronounced for developing countries where students join medical school at a much younger age than their Global North counterparts [11, 12] and may need to grapple with a unique set of barriers to online learning. By examining these challenges within the specific context of Pakistan, this study aimed to examine the psychosocial and cognitive impact of online learning on medical students during and after the COVID-19 pandemic, and explore their perceptions of the effectiveness of various online-learning pedagogies. By doing so, this study sought to provide actionable insights into how online education can be optimized to support medical students’ learning and well-being.

## Methods

### Study design and ethical considerations

We conducted an explanatory sequential mixed-methods study including medical students from various medical colleges in Karachi, Pakistan from July 2021 to February 2022 after obtaining ethical approval from the AKU institutional review board (ERC#2021-5956-17622). Using an explanatory sequential study design enabled us to not only identify general trends, but also deeply explore students’ nuanced perceptions and experiences based on those trends. The quantitative phase was a cross-sectional descriptive study conducted digitally by a questionnaire completed by medical students enrolled at AKU and other medical schools located in Karachi, Pakistan, followed by a qualitative phase using an exploratory phenomenological design with semi-structured focus group discussions (FGDs) to explore the validity of our quantitative findings. Informed consent was obtained from all study participants. Authorship was determined using a published rubric [13].

### Eligibility criteria

The inclusion criteria comprised all medical students currently enrolled in a five-year medical school curriculum across Karachi who self-reported having engaged in entirely online medical learning for a continuous period of at least six months, either prior to or during the study period (July 2021 to February 2022). Students on extended leave for any reason were excluded from the study. To broaden the scope of the study and capture a more diverse set of experiences with online medical education, students from other medical colleges in Karachi were also invited to participate.

### Sample size calculation

For the quantitative part of this study, we calculated a minimum sample size of 264, assuming a precision level of 5%, an anticipated rate of dissatisfaction with online learning of 78% based on a similar study on online learning for medical students conducted in Pakistan in 2020, and a confidence interval of 95% [14].

For the qualitative part of the study, we invited students from Years II to V enrolled at AKU to take part in FGDs. Year I students were excluded given that they did not meet the eligibility criterion of having been engaged in at least 6 months of entirely online learning at the time the study was conducted. A minimum of two FGDs were deemed appropriate to obtain an adequately diverse range of perspectives and to achieve data saturation based on previous literature [15].

### Data collection tool and procedure

#### Quantitative– digital questionnaire

The study team self-designed a questionnaire after an intensive literature review, with components adapted (with permission) from Leong et al. (2011) [16]. The questionnaire was developed in English, given that English is the primary language of instruction in medical schools across Pakistan. To ensure validity, the questionnaire was reviewed for face validity by experts in the field, confirming that it appropriately measured the intended constructs. We also conducted a pilot test on 10 medical students from AKU to rectify errors. Purposive and snowball sampling techniques were used to disseminate the digital survey via email and social media platforms including WhatsApp, Facebook, and Instagram.

In addition to demographic information, the questionnaire had four sections with responses using a Likert scale. We added both positive and negative statements to minimize response bias in the non-demographic sections of the survey. Furthermore, participants were provided with the option to select “Not applicable/Not experienced” for each teaching method listed in the questionnaire. This allowed students to indicate when a particular modality was not offered at their institution or when they had not personally experienced it.

1. *Computer Usage*: Included questions about their computer usage for academic, entertainment, and social purposes as well as the quality of internet connectivity they had available to them.
2. *Cognitive Effects*: Included questions pertaining to students’ level of concentration with different teaching pedagogies, focus, critical thinking, knowledge-retention, and self-regulation abilities.
3. *Social Effects*: Included students’ relationship with their classmates and teachers, and its impact on communication and interactions during learning.
4. *Psychological Effects*: Asked students to rate the impact of online learning on psychological parameters including anxiety, quality of sleep, social stress, mental well-being, and academic motivation.

#### Qualitative– focus group discussion

An independent, trained interviewer conducted two in-person, semi-structured FGDs with students recruited via convenience sampling from our institute who responded to the initial quantitative survey. As such, integration of the study phases through a methods-level connecting approach was achieved, which was deemed to be most appropriate for our explanatory sequential study design [17]. The investigators ensured that there was equal representation of gender and year of study. An interview guide based on the general trends and findings of the quantiative phase of the study was prepared beforehand and approved by the study team to glean as much relevant information from the participants as possible. The interview guide consisted of questions that encouraged participants to compare and contrast the cognitive, psychological, and social impacts of FTF learning with online learning, including the different subtypes of each. More specifically, prompts included encouraging participants to share their perspectives on how online learning affects their mental and emotional well-being, their ability to absorb and retain knowledge, potential improvements to online learning formats to better support both academic success and personal well-being, and an opportunity to provide any additional comments not otherwise mentioned in the discussion. Each FGD lasted 60 min and was audio-recorded, with data saturation being achieved after two interviews with a total of 18 students (9 students per FGD). The interviewer took field notes to process-capture non-verbal cues, emotional expressions and physical actions or reactions of participants during the interview process.

### Data analysis

#### Statistical analysis– quantitative

Statistical analyses were run using International Business Machines (IBM) Statistical Package for Social Sciences (SPSS) version 24. All continuous data were non-parametrically distributed and thus represented as medians and interquartile ranges (IQR) while categorical data were represented as frequencies and percentages. Comparisons between categorical variables were conducted using Pearson’s 𝜒^2^ test. A *p*-value < 0.05 was considered as significant for all analyses.

Additionally, subgroup comparisons were conducted between AKU and non-AKU student responses for key survey items. Although the small sample size of the non-AKU group limited the statistical power of these comparisons, no meaningful or consistent differences in response trends were identified. Therefore, the data from all respondents were aggregated and reported collectively to reflect the overall findings.

#### Thematic analysis– qualitative

The FGD was transcribed verbatim in English by the research team and reviewed for quality by all investigators. The transcription was also sent to all participants for member checking and agreement, after which the transcribed data was de-identified to maintain confidentiality. The scissors-and-sort technique was used to code the data on Microsoft Excel by two independent members of the study team, after which relevant sub-themes and themes were derived from the data based on consensus. The analysis used a thematic approach, where the existence and repetition of themes and concepts in the text were considered crucial factors in their consideration in interviewee responses. All relevant information was presented in narrative fashion and in descriptive memoranda. Reporting-level integration between study phases was achieved by integrating through narrative, more specifically using the contiguous approach in the results and the weaving approach in discussion sections of the manuscript, respectively.

## Results

### Demographics and screentime

A total of 262 medical students responded to the survey, of which 227 (86.6%) were enrolled at the Aga Khan University (AKU) and 35 (13.4%) were from two other medical colleges in Karachi. At the time of data collection, the total enrollment across all five years of the MBBS program at AKU was approximately 500 students, yielding a participation rate of ~ 45% for AKU students. There was an approximately even distribution of males and females (46.9% and 52.3%, respectively), and pre-clinical and clinical (51.1% and 48.9%, respectively) students. Over half of the respondents were day scholars (57.6%). Academic screentime increased significantly due to the COVID-19 pandemic. Before the pandemic, over half of the respondents (52.3%) reported spending less than one hour on academic screentime daily, compared to only 5% during the pandemic (*p* < 0.001). Most students (80.5%) spent > 3 h of screentime for academic purposes during/after the COVID-19 pandemic. These results are represented in Table 1.

**Table 1:** Medical students’ demographic characteristics and screentime *N* = 262

Variable	N (%)/Median (IQR)
**Age (in years)**	21 (20–22)
**Year of Study**	
Year I	86 (32.8)
Year II	48 (18.3)
Year III	63 (24.0)
Year IV	44 (16.8)
Year V	21 (8.0)
**Gender**	
Male	123 (46.9)
Female	137 (52.3)
Prefer not to say	2 (0.8)
**Student Status (Day Scholar/Hostelite)**	
Day Scholar	151 (57.6)
Hostelite	111 (42.4)
**Country of Origin**	
Pakistan	246 (93.9)
Other	16 (6.1)
**Age at which first personal device (computer, tablet, smartphone) was used (in years)**	10 (7–12)
**Daily screentime for gaming**	
< 1 hour	215 (82.1)
1–2 hours	31 (11.8)
3–4 hours	10 (3.8)
≥ 5 hours	6 (2.3)
**Daily screentime for other entertainment**	
< 1 hour	29 (11.1)
1–2 hours	98 (37.4)
3–4 hours	90 (34.4)
≥ 5 hours	45 (17.2)
**Daily screentime for academic purposes before the COVID-19 pandemic**	
0–1 hours	137 (52.3)
1–2 hours	64 (24.4)
3–4 hours	37 (14.1)
≥ 5 hours	24 (9.2)
**Daily screentime for academic purposes during/after the COVID-19 pandemic**	
0–2 hours	13 (5.0)
1–2 hours	38 (14.5)
3–4 hours	91 (34.7)
≥ 5 hours	120 (45.8)
**Had adequate quality of connectivity during online sessions**	
Agree/Strongly Agree	127 (48.5)
Disagree/Strongly Disagree	90 (34.4)

### Cognitive, social and psychological impacts of online learning

#### Gender-based differences in online learning

Male students felt less engaged by facilitators during online sessions (52.8% vs. 32.1%; *p* = 0.007), while female students did not find online sessions helpful in strengthening their concepts (26.3% vs. 13.8%; *p* = 0.035). Female students also reported less collaborative learning during online sessions (32.1% vs. 17.1%; *p* = 0.002), felt less motivated to study (37.2% vs. 21.1%; *p* = 0.007), and were less likely to have reduced anxiety about learning performance (40.1% vs. 26.8%; *p* = 0.042).

#### Duration of online exposure and learning outcomes

Students who spent less time learning online during the COVID-19 pandemic were more likely to feel that online learning improved their self-regulation skills. Specifically, 53.8% of students who spent less than one hour learning online daily reported this benefit, compared to only 25.8% of those who spent more than four hours (*p* = 0.019). Moreover, students who spent more time online for entertainment were more likely to disagree that online learning helped achieve module/clerkship objectives (28.9% in those spending > 4 h for online entertainment vs. 17.2% for those spending < 1 h; *p* = 0.019) or that they were able to retain knowledge from online sessions (33.3% in > 4 h vs. 20.7% in < 1 h; *p* = 0.014). The duration of time spent on daily online learning during the pandemic, when dichotomized to greater than 4 h and less than or equal to 4 h, was significantly associated with self-reported mental exhaustion (*p* = 0.001), but had no signficant impact on other learning, social, or psychological outcomes.

#### Clinical vs. pre-clinical differences in online learning

Only 17.2% of students felt most facilitators kept them engaged during online sessions, while 42.0% disagreed, with pre-clinical students reporting this more frequently than clinical students (49.3% vs. 34.4%; *p* = 0.038). While pre-clinical students were more likely to disagree that online learning reduces social stress (50.0% vs. 36.7%; *p* = 0.003), a greater proportion of them disagreed they felt physically (47.8% vs. 27.3%; *p* = 0.001) and mentally (61.2% vs. 40.6%; *p* = 0.002) exhausted at the end of the workday compared to their clinical colleagues, and were less likely to have trouble paying attention during online studies (*p* = 0.007). Although both groups of students were less likely to agree that they could engage with their peers in online sessions (31.3% vs. 36.3%), a higher proportion of both groups of students felt that online sessions strengthened their critical thinking (49.6% vs. 14.9%), helped achieve relevant module/clerkship objectives (33.6% vs. 24.4%), allowed for collaborative learning (48.5% vs. 24.8%), helped build student facilitator relationships (58.0% vs. 16.4%) and improved their sense of belonging (51.1% vs. 22.5%). Additional comparisons have been reported and summarised in Table 2.

**Table 2:** Cognitive, social, and psychological impact of online learning on medical Students*Responses marked as neutral are not shown in this table

Question	Total (N = 262)	Pre-Clinical (N = 134)	Clinical (N = 128)	*P*-Value
	n (%)	n (%)	n (%)	
Most facilitators helped us stay engaged during online sessions				0.038
Agree/Strongly Agree	45 (17.2)	18 (13.4)	27 (21.1)	
Disagree/Strongly Disagree	110 (42.0)	66 (49.3)	44 (34.4)	
**Online sessions strengthened key concepts**				**0.016**
Agree/Strongly Agree	111 (42.4)	58 (43.3)	53 (41.4)	
Disagree/Strongly Disagree	53 (20.2)	35 (26.1)	18 (14.1)	
**Online sessions strengthened critical thinking**				0.536
Agree/Strongly Agree	130 (49.6)	66 (49.3)	64 (50.0)	
Disagree/Strongly Disagree	39 (14.9)	23 (17.2)	16 (12.5)	
**Online sessions help achieve relevant module/clerkship objectives**				0.392
Agree/Strongly Agree	88 (33.6)	41 (30.6)	47 (36.7)	
Disagree/Strongly Disagree	64 (24.4)	37 (27.6)	27 (21.1)	
**Able to retain learning from online sessions**				0.103
Agree/Strongly Agree	91 (34.7)	41 (30.6)	50 (39.1)	
Disagree/Strongly Disagree	88 (33.6)	53 (39.6)	35 (27.3)	
**I had trouble staying focused during online studies**				**0.007**
Agree/Strongly Agree	44 (16.8)	24 (17.9)	20 (15.6)	
Disagree/Strongly Disagree	158 (60.3)	90 (67.2)	68 (53.1)	
**Online sessions allow easy communication**				0.388
Agree/Strongly Agree	99 (37.8)	56 (41.8)	43 (33.6)	
Disagree/Strongly Disagree	91 (34.7)	44 (32.8)	47 (36.7)	
**I was able to engage with peers during online sessions**				0.646
Agree/Strongly Agree	82 (31.3)	40 (29.9)	42 (32.8)	
Disagree/Strongly Disagree	95 (36.3)	47 (35.1)	48 (37.5)	
**Online sessions allow collaborative learning**				0.751
Agree/Strongly Agree	127 (48.5)	68 (50.7)	59 (46.1)	
Disagree/Strongly Disagree	65 (24.8)	32 (23.9)	33 (25.8)	
**Online learning helped build student-facilitator relationships**				0.477
Agree/Strongly Agree	152 (58.0)	78 (58.2)	74 (57.8)	
Disagree/Strongly Disagree	43 (16.4)	25 (18.7)	18 (14.1)	
**Online learning has helped improve my sense of responsibility**				**0.008**
Agree/Strongly Agree	123 (46.9)	62 (46.3)	61 (47.7)	
Disagree/Strongly Disagree	56 (21.4)	38 (28.4)	18 (14.1)	
**Online learning has helped improve my sense of belonging**				0.653
Agree/Strongly Agree	134 (51.1)	68 (50.7)	66 (51.6)	
Disagree/Strongly Disagree	59 (22.5)	33 (24.6)	26 (20.3)	
**Online sessions have helped improve my academic performance**				0.634
Agree/Strongly Agree	109 (41.6)	53 (39.6)	56 (43.8)	
Disagree/Strongly Disagree	53 (20.2)	30 (22.4)	23 (18.0)	
**Online learning reduces social stress**				**0.003**
Agree/Strongly Agree	91 (34.7)	49 (36.6)	42 (32.8)	
Disagree/Strongly Disagree	114 (43.5)	67 (50.0)	47 (36.7)	
**Online sessions help improve my sleep schedule**				**0.003**
Agree/Strongly Agree	81 (30.9)	50 (37.3)	31 (24.2)	
Disagree/Strongly Disagree	130 (49.6)	68 (50.7)	62 (48.4)	
**Online sessions help improve my motivation to self-learn/self-study**				0.101
Agree/Strongly Agree	108 (41.2)	57 (42.5)	51 (39.8)	
Disagree/Strongly Disagree	77 (29.4)	45 (33.6)	32 (25.0)	
**Online sessions reduced my anxiety about learning performance**				**0.006**
Agree/Strongly Agree	96 (36.6)	55 (41.0)	41 (32.0)	
Disagree/Strongly Disagree	88 (33.6)	51 (38.1)	37 (28.9)	
**Online learning has reduced my sense of well-being**				**0.05**
Agree/Strongly Agree	91 (34.7)	52 (38.8)	39 (30.5)	
Disagree/Strongly Disagree	64 (24.4)	37 (27.6)	27 (21.1)	
**Online sessions helped learn self-regulation**				**0.001**
Agree/Strongly Agree	81 (30.9)	37 (27.6)	44 (34.4)	
Disagree/Strongly Disagree	96 (36.6)	63 (47.0)	33 (25.8)	
**Feel physically exhausted during online classes**				**0.001**
Agree/Strongly Agree	97 (37.0)	46 (34.3)	51 (39.8)	
Disagree/Strongly Disagree	99 (37.8)	64 (47.8)	35 (27.3)	
**Feel mentally exhausted and drained at the end of the day**				**0.002**
Agree/Strongly Agree	75 (28.6)	34 (25.4)	41 (32.0)	
Disagree/Strongly Disagree	123 (51.1)	82 (61.2)	52 (40.6)	

### Medical students’ concentration with different teaching pedagogies

The ability of medical students to maintain concentration during online sessions varied depending on the teaching method employed. Most students (68.3%) reported staying focused during online PBL sessions, while online laboratory sessions were the most challenging, with only 24.0% agreeing that they were able to focus. Results for additional teaching modalities are represented in Table 3.

**Table 3:** Medical students’ concentration with different teaching Pedagogies*Responses marked as neutral are not shown in this table

Question	N (%)
**Were able to stay focused during online PBL sessions.**	*N = 230*
Agree/Strongly Agree	157 (68.3)
Disagree/Strongly Disagree	51 (22.2)
**Were able to stay focused during online TBL sessions?**	*N = 225*
Agree/Strongly Agree	88 (39.1)
Disagree/Strongly Disagree	85 (37.8)
**Were able to stay focused during online laboratory sessions?**	*N = 225*
Agree/Strongly Agree	54 (24.0)
Disagree/Strongly Disagree	122 (54.2)
**Were able to stay focused during online lectures?**	*N = 258*
Agree/Strongly Agree	105 (40.7)
Disagree/Strongly Disagree	110 (42.6)
**Were able to stay focused during online clinical skills sessions?**	*N = 236*
Agree/Strongly Agree	141 (59.7)
Disagree/Strongly Disagree	62 (26.3)
**Were able to stay focused during online tutorials?**	*N = 251*
Agree/Strongly Agree	122 (48.6)
Disagree/Strongly Disagree	80 (31.9)
**Were able to stay focused during online teleclinics?**	*N = 168*
Agree/Strongly Agree	69 (41.1)
Disagree/Strongly Disagree	44 (26.2)

### Focus group discussion– thematic analysis results

**Fig. 1:**
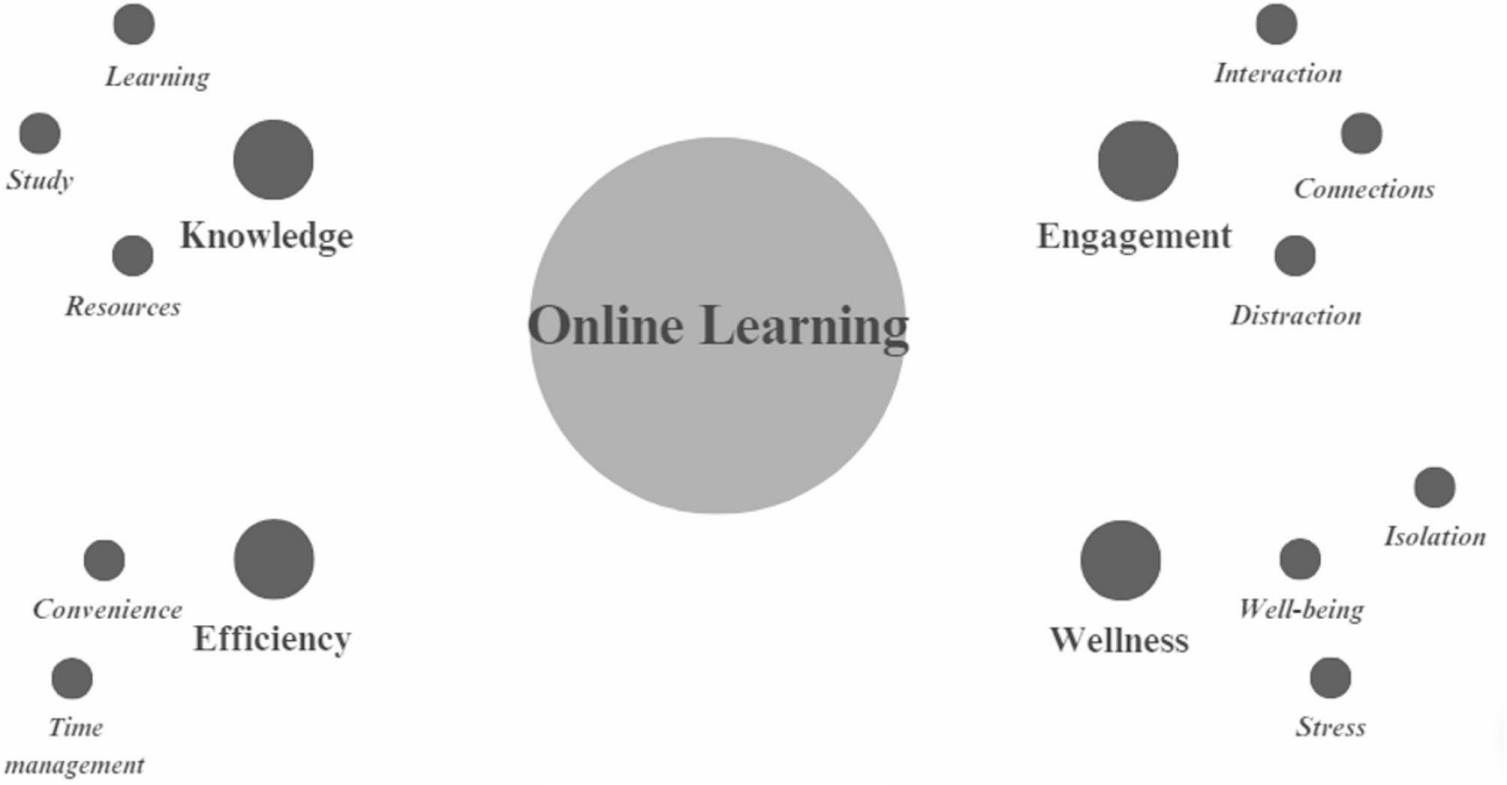
Themes and sub-themes generated through qualitative analysis. *Caption*: All themes from the qualitative analysis are represented by the larger dark gray circles, while subthemes are represented by the smaller dark gray circles

A total of 18 students from Year II to Year V participated in two semi-structured FGDs. Participants were asked to describe the advantages and disadvantages of online learning compared to FTF learning, especially regarding cognitive and psychosocial aspects. The following themes (each with sub-themes) were generated: Efficiency, Knowledge, Engagement and Wellness **(**Fig. 1**).**

#### Efficiency

Convenience was described as one of the major advantages of online learning, especially recorded lectures allowed revisitation of content, reviewed at higher speeds, and covered in shorter duration, thereby preventing students from *“zoning out”* in comparison to longer FTF sessions. According to one student, “*Recorded lectures give you the liberty to study at your own pace; you can always go back to them and review”*. Some students felt that in live, online lectures, it is easier to search and answer questions, though retention was poor. Time spent commuting and getting ready could be utilized for activities such as exercise, thereby allowing a better study-life balance. Additionally, resources can be swiftly shared with the class using the chat function of video conferencing platforms like Zoom and Microsoft Teams.

#### Engagement

Several students mentioned that it was easier to get distracted in online learning by virtue of being in the comfort of their homes, lying down, or *“tabbing out”* on their laptops to use social media/entertainment platforms. Conversely, students felt that *“zoning out”* was not specific to online or FTF modalities, but up to the student to concentrate and session interactivity. In online lectures, some students found inquiry and engagement difficult and felt teachers were unable to gauge students’ comprehension. A student stated, ‘*someone that’s in-person can manage the energy of an entire classroom*,* and bring students in who weren’t even that excited about the lecture’*. Students also described a variety of digital access issues, including the dependence of online learning on access to a digital device with a camera, stable and reasonably fast internet connection, and a reliable electricity supply, which may preclude all students from enjoying the benefits of online learning equally. As such, some students perceived that the quality of the online learning experience was more influenced by individual circumstances than was the case with FTF teaching.

#### Knowledge

According to several students, some subjects may be better taught FTF, like anatomy, while others like microbiology, biochemistry and physiology can be taught online to some extent. However, using videos, 3D atlases, and high-quality animations can make understanding of anatomy and embryology better for some students in the online format. Students also felt that some teaching strategies, like lectures, may be better suited for online, whereas others, like PBLs, should be FTF. Ultimately, most students agreed that whether a session should be online or FTF is largely a matter of personal preference.

#### Wellness

Many students felt their ability to socialize was compromised with online learning. According to one student, ‘*it’s just academics without the other part of it*,* you know*,* your whole wellbeing*,* meeting people*,* discussing your problems*,* you don’t have that online*,* especially when you’re adjusting to a new place like AKU’*. However, other students felt that communicating with each other on WhatsApp groups or Discord servers was a positive change. Some students felt isolated online, and agreed that this can significantly impact one’s learning. As one student stated, ‘*face to face learning creates a better bond between the people who are studying’.* For example, online learning makes interacting with one’s seniors far more difficult, especially for those who have none of their high school seniors in their university. As such, informal guidance that one would otherwise receive from seniors, such as how much to read and the best resources to use, becomes relatively inaccessible with online learning, thereby leading to stress and anxiety. At the end one student stated, ‘*for me*,* personally*,* an ideal balance will be somewhere in between’* online and FTF teaching.

## Discussion

Our mixed-methods explanatory sequential study, encompassing medical students at AKU and other medical education programs located in Karachi, Pakistan provides valuable insights into the multifaceted impacts of a transition to online learning, and explores the opinions of students on online modalities compared to traditional FTF methods. As electronic pedagogy persists in a post-pandemic era, the psychosocial and cognitive aspects of online education warrant exploration in medical education, where learning is largely dependent on social constructivism.

Our study saw a significant shift in screen time for academic purposes during and since the pandemic, which is consistent with global trends where educational institutions have rapidly transitioned to online platforms since the pandemic [18, 19]. This digital immersion, combined with screen time for entertainment, underscores a profound change in the daily routines of medical students. Although the levels of screentime during the pandemic may not be comparable with those in a post-pandemic era, there are some key insights about online learning that can be extrapolated. For example, the potential for digital “zoom” fatigue and the associated challenges of students’ mental health and maintaining concentration cannot be overlooked [20]. A majority of students felt that they had trouble concentrating in online sessions, which, as per the FGDs, may be attributable to a lack of accountability, family distractions, and the ability to “tab out” and do something else on their devices.

Moreover, we found that a lack of sustained attention, focus, self-monitoring and regulation, and time management were more significant in junior students. This is similar to the findings of a previous systematic review on school performance among children and adolescents during the pandemic which suggests that younger students face more difficulty concentrating on online platforms [21]. Given that medical students in Pakistan are relatively younger than their Western counterparts and are chronologically closer to high school students, this may explain why junior medical students struggled more in the cognitive domain in our study [11, 12]. Notably, in contrast to prior research, our findings also reveal that female students faced difficulties in assimilating concepts during online sessions [5, 22]. This observation aligns with earlier studies suggesting that female medical students are more likely to be kinesthetic learners [23].

Besides this, online learning was not seen to have a major benefit on reducing students’ anxiety about learning performances, and almost one-third of students agreed that online sessions had reduced their sense of well-being. This may be attributable to compromised social interactions and an associated feeling of isolation. As per the FGDs, the absence of the social fabric of traditional learning—a space for informal interactions, peer discussions, and group studies—seemed to be a significant drawback of online learning, especially for students in their earlier years of medical school. This social dimension, often overlooked, plays a crucial role in alleviating academic stress, fostering collaborative learning, and building a sense of community [24–26]. Online learning may also be associated with sleep disturbances [27], which is reflected by our results where approximately half of the participants disagreed that online learning had improved their sleep schedule. These drawbacks have significant implications for curriculum planning, emphasizing the need for balanced screen exposure, and potentially incorporating breaks or offline activities to counteract digital fatigue and improve mental well-being.

On the other hand, in moderation, online learning modalities may offer significant benefits. Students in our study with less screentime exposure were less likely to have trouble staying focused in online sessions and were more likely to feel that online learning helped them learn self-regulation. Our results also displayed that students felt online platforms were more convenient than FTF learning due to a lack of commute, increased accessibility, and content being available to review and revisit as per one’s convenience. In addition, a higher proportion of students were seen to agree that online learning had strengthened key concepts and critical thinking abilities, encouraged collaborative learning, and even improved academic performance. These results are in agreement with existing literature and emphasize that online teaching modalities have the potential to substantially benefit student learning if used well [3, 28].

However, the effectiveness of online learning for students depends on a multitude of factors. One of these is how effectively online modalities can be used by educators [29]. Inexperience with online pedagogies can impact the delivery of the content teachers are accustomed to delivering in-person, as highlighted by the FGD, and may also lead to less engaging and interactive classes. This is shown by how a vast majority of participants felt that most of their facilitators did not help them stay engaged in sessions, many of whom were using online modalities for the first time. These findings are concurrent with other studies conducted in Pakistani medical colleges that report rates of dissatisfaction with online learning of up to 78% which were majorly attributable to difficulties in meaningfully engaging with their instructors online [14, 30]. This underscores the importance of the facilitator’s role in making online sessions interactive, engaging, and relevant. Their ability to adapt content to the digital format, use multimedia effectively, and foster an inclusive, supportive, and interactive virtual classroom atmosphere can significantly impact the online learning experience. As such, incorporating online learning into medical education necessitates investing in training and support for medical educators for them to better utilize online teaching modalities.

The effectiveness of online learning is also significantly influenced by individual student circumstances, particularly stable internet access, as highlighted by the FGD. Unlike FTF teaching, where students share a uniform environment, online learning varies widely, potentially leading to isolation, reduced social interaction, and limited emotional support. Disparities in technological access can exacerbate inequities, disadvantaging students from lower socioeconomic backgrounds and affecting their well-being and academic confidence. However, with adequate support, these challenges can also foster adaptability and resilience. Addressing these psychosocial impacts is essential for creating equitable and effective online learning environments.

In addition, the psychosocial implications of online learning modalities, as revealed by our study, highlight a complex interplay between pedagogy, content, and student engagement. We found that interactive pedagogies, such as PBLs and online clinical skills sessions, which require active participation, were effective in maintaining student focus and engagement, contributing to a more positive student experience. Conversely, more didactic approaches, such as traditional lectures and laboratory sessions, were perceived as less engaging when delivered live but were deemed more suitable for recording. This adaptability allowed students to control their learning pace by revisiting content and adjusting playback speed, catering to their individual learning styles and potentially reducing stress associated with real-time comprehension. Content that lends itself well to visual and interactive enhancements, such as microbiology, biochemistry and physiology, was identified as particularly amenable to online formats. These subjects, which rely heavily on conceptual understanding, can be effectively conveyed through digital tools and animations. However, areas like gross anatomy and certain clinical skills, which depend on hands-on practice and direct patient interaction, presented significant challenges in an online setting. The depth and nuances of patient interactions that are experienced in-person in clinics, the operating room, or in-patient wards are what underpin clinical skill acquisition and as such, online modalities may exist to complement such learning but can never truly substitute it entirely [2, 10].

### Limitations

Our study is bound by some limitations. The geographical specificity of our study limits the generalizability of our conclusions to the rest of Pakistan. Moreover, given our participants were predominantly from one private medical college i.e. AKU, the findings may disproportionately reflect the experiences of students at a single, relatively well-resourced institution, potentially limiting the generalizability of results to settings with fewer resources or different curricular structures. Our generalizability is also impacted by the fact that the minimum sample size calculation was done based on a different primary outcome (used as a proxy) from an existing study at the time this study was conceived. Furthermore, the cross-sectional design of this study does not reflect the evolving nature of students’ experiences. Additionally, self-reported data are subject to recall and social desirability biases, and the study did not independently verify institutional practices or policies. Besides this, the survey instrument was not tested for internal consistency reliability, such as through Cronbach’s alpha, which limits the ability to assess the coherence of responses across related items measuring similar constructs. Lastly, while the survey captured a minimum six-month period of online learning, students’ experiences may have varied based on the specific timing and intensity of online instruction during the COVID-19 pandemic. Despite these limitations, the findings of this mixed-methods study can serve as a springboard to generate discourse on the refinement of online learning strategies to optimize medical education for students in a post-pandemic era in similar settings.

### Future research

Future research should include diverse regions and participants from both public and private medical colleges in Pakistan to improve generalizability. Larger, well-calculated samples and longitudinal designs are needed to track changes in perceptions, academic performance, clinical skills, and mental health over time. Studies on hybrid learning models can identify optimal approaches for integrating online and face-to-face education, especially in a post-pandemic era. In addition, further investigation into faculty perspectives and the role of innovative technologies like virtual reality and AI-driven simulations is essential regarding online learning. Lastly, research should address equity and accessibility, focusing on how socioeconomic and technological factors impact online learning outcomes.

## Conclusions

A shift to online learning in medical education presents both opportunities and challenges, with clear cognitive and psychosocial effects. Our findings suggest that while online learning can be a valuable adjunct in medical education, a hybrid, student-centric model, combining the best of both online and FTF modalities, might be the best way forward. Such a model would allow medical students to benefit from the flexibility and accessibility of online platforms while preserving the invaluable social and interactive aspects of traditional learning, thereby helping to address students’ fundamental mental, social, emotional, and motivational needs. Moreover, as medical education continues to evolve, there is a need for a more holistic approach, one that not only imparts clinical knowledge and skills but also addresses the well-being and mental health of students for which online learning, in moderation, may be beneficial.

## Data Availability

The datasets used and/or analysed during the current study are available from the corresponding author on reasonable request.
